# Supplementary figures and images for: Gut Microbial Composition and Liver Metabolite Changes Induced by Ammonia Stress in Juveniles of an Invasive Freshwater Turtle
Source: Biology (Basel). 2022 Sep 5;11(9):1315. doi: 10.3390/biology11091315 (PMC9495491; doi:10.3390/biology11091315)

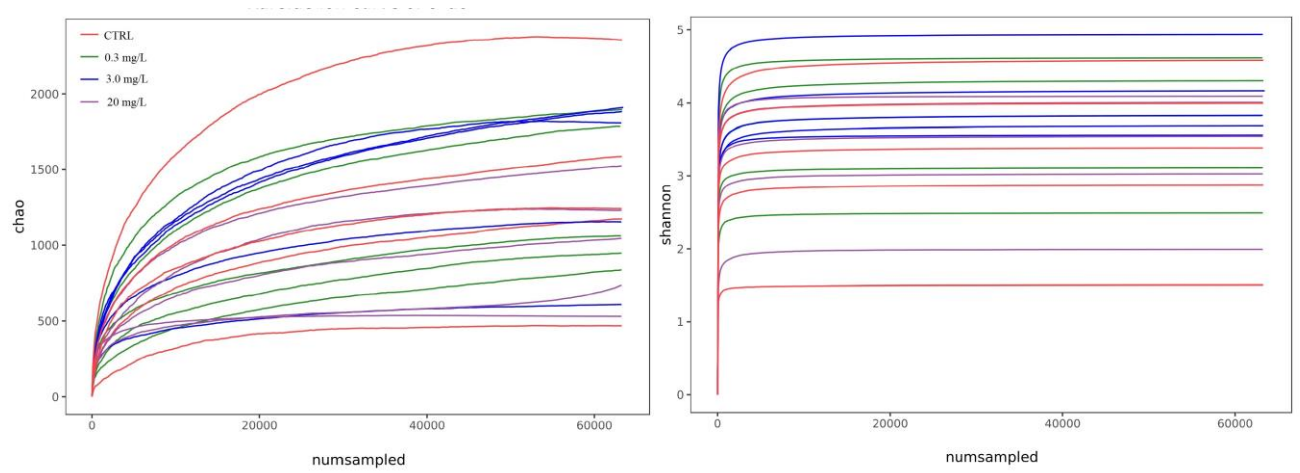

Figure S1: Rarefaction curves of Chao and Shannon--Wiener indexes for all samples.

Supplement: Supplementary file 1 [file biology-11-01315-s001.zip › biology-1887765-supplementary.pdf]
